# Pharmacological manipulation of AID

**DOI:** 10.18632/oncotarget.5269

**Published:** 2015-08-26

**Authors:** Stephen P. Methot, Javier M. Di Noia

**Affiliations:** Institut de Recherches Cliniques de Montréal, Department of Medicine, McGill University, Department of Medicine, Université de Montréal, Montréal, QC, Canada

**Keywords:** activation induced deaminase, HSP90, eEF1A, antibody diversification, oncogenic activity

In order to have an efficient humoral immune response, activated B lymphocytes with weak B cell receptor affinity for cognate antigen must undergo secondary antibody diversification. They do this using the mechanisms of somatic hypermutation (SHM), to boost the affinity of their antigen recognition domain, and class switch recombination (CSR), to exchange their effector domain, which specifies function. SHM and CSR are initiated by the enzyme Activation induced deaminase (AID), which transforms DNA deoxycytidine into deoxyuridine at the immunoglobulin (*Ig*) genes. A proportion of these mutagenic lesions are processed into other point mutations or DNA breaks to underpin antibody diversification.

The lack of functional AID causes Hyper-IgM type 2; an immunodeficiency syndrome caused by the absence of SHM and CSR. On the other hand, off-target AID activity can be pathogenic by mutating outside the *Ig* loci. Indeed, disregulated AID is linked to autoimmune diseases as well as oncogenesis, cancer progression and chemoresistance. Several mechanisms regulate AID and can limit its pathogenic potential. While the need for these mechanisms is clear, measuring their distinct impact on AID activity can be challenging. This complexity is nicely illustrated by the regulation of AID protein stability and subcellular localization. Even though the physiological target of AID is inside the nucleus, several mechanisms promote a predominantly cytoplasmic localization. First, active nuclear import of AID is counteracted by a mechanism of cytoplasmic retention [1], and nuclear AID is exported back to the cytoplasm via the CRM1 carrier, so that a fraction of AID constantly shuttles between these two compartments [2]. Second, cytoplasmic AID has a significantly longer half-life than nuclear AID [3, 4] because the HSP90 molecular chaperoning pathway protects cytoplasmic AID [3] while a fraction of nuclear AID is targeted for rapid proteasomal degradation [4].

**Figure 1:**
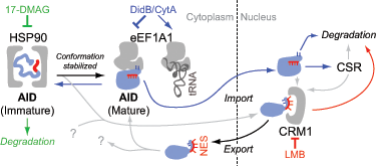
Model for AID maturation and localization Different conformations of AID are illustrated with the nuclear export signal in red and residues mediating retention in blue. Colour-coded arrows show the different outcomes of inhibitor treatments. Grey arrows indicate unverified connections.

Our recent characterization of AID cytoplasmic retention and the identification of drugs that can inhibit it allowed us to determine its biological relevance, and to highlight differences with other regulatory mechanisms [5]. Cytoplasmic retention of AID is mediated by a conserved motif within its C-terminal domain [1]. Our results are consistent with this region adopting a specific conformation in order to expose certain residues that mediate retention (Figure 1). The efficiency with which different AID variants were retained in the cytoplasm correlated perfectly with their relative ability to interact with the translation elongation factor eEF1A1, which had been previously shown to associate with cytoplasmic AID [6].

To test the potential role for eEF1A1 in AID cytoplasmic retention we could not use genetic ablation, as eEF1A1 is essential for cellular survival because it transports aminoacyl tRNA to the ribosome during mRNA translation. Consequently, we used two different inhibitors of translation elongation that blocked eEF1A1 function; Didemnin B and Cytotrienin A. Both drugs disrupted the interaction of AID with eEF1A1 and concomitantly stabilized eEF1A1 binding to tRNA, suggesting a mutually exclusive interaction (Figure 1). This was accompanied by an increase in the fraction of nuclear AID and in its biological activity, in the form of both CSR and oncogenic *IgH-cMyc* chromosomal translocations. Appropriate controls indicated that this effect is independent of eEF1A1 activity in mRNA translation. Thus, although the AID-eEF1A1 complex likely contains other proteins, eEF1A1 is a necessary component for retaining AID. More importantly, it limits overall AID activity by sequestering a pool of mature AID in the cytoplasm.

Only the simultaneous inhibition of eEF1A1 and CRM1 resulted in full nuclear accumulation of endogenous AID, while inhibiting each separately had much smaller effects on AID localization. Thus, cytoplasmic retention and nuclear export cooperate in limiting the amount of nuclear AID. The previous interpretation that AID nuclear export was the major force limiting its nuclear access [2] was based on the analysis of GFP-tagged AID and mutations in the nuclear export signal. Both these alterations would affect the AID conformation necessary for its cytoplasmic retention, explaining the apparent discrepancy (Figure 1). Interestingly, CRM1 inhibition did not cause any observable increase in CSR, suggesting the possibility that it may export a biologically inactive pool of AID (Figure 1).

The relative binding of AID variants to eEF1A1 was also inversely correlated with their binding to HSP90. Since HSP90 stabilizes metastable proteins, this suggests a step-wise maturation of AID whereby HSP90 promotes folding of an immature form of AID into the conformation that then binds to eEF1A1 (Figure 1). Indeed, HSP90 inhibitors cause AID degradation and reduce its mutagenic potential [3], the opposite outcome to inhibiting eEF1A1.

The identification of drugs that can modulate AID activity opens up a new avenue for therapeutics. HSP90 inhibitors are well-characterized drugs currently in clinical trials against cancer and autoimmune disease. We recently showed that the HSP90 inhibitor 17-DMAG is able to lower AID levels *in vivo*, which reduced antibody diversification as well as some tumour promoting effects of AID [7]. The eEF1A1 inhibitors could work in the opposite sense, by boosting AID activity *in vivo*. This would be particularly useful for the generation of therapeutic antibodies such as highly mutated broadly neutralizing antibodies or for the elderly, whose immune system and vaccine response are weak, in part due to reduced AID expression. Didemnin B was tested as a cytotoxic agent in cancer patients but was abandoned due to high toxicity. However, low Didemnin B doses might sufficiently impair the AID-eEF1A1 interaction to be used as an adjuvant following immunization; thus mitigating its toxicity and the potential oncogenic side effects of AID. Alternatively, development of less toxic drugs will be necessary.

AID is a clear example of the immune system juggling its protective response with dangerous side effects. Equilibrium is maintained by many regulatory mechanisms, each one impacting differently on AID physiological and pathological activities. Defining the molecular basis for AID stabilization and cytoplasmic retention has provided tools to manipulate this equilibrium.
